# Histoplasmosis in Taiwan: Case Summary and Literature Review

**DOI:** 10.3390/life14060738

**Published:** 2024-06-07

**Authors:** Jui-Chi Hsu, Po-Hsun Chang, Chien-Hsiang Tai, Yi-Chun Chen

**Affiliations:** 1 Division of Infectious Diseases, Department of Internal Medicine, Kaohsiung Chang Gung Memorial Hospital, Kaohsiung 83301, Taiwan; b9602048@cgmh.org.tw (J.-C.H.); b9702096@cgmh.org.tw (C.-H.T.); 2 Department of Pharmacy, Kaohsiung Chang Gung Memorial Hospital, Kaohsiung 83301, Taiwan; pohsunchang@cgmh.org.tw; 3 Department of Public Health, College of Health Science, Kaohsiung Medical University, Kaohsiung 80708, Taiwan; 4 College of Medicine, Chang Gung University, Taoyuan 33302, Taiwan

**Keywords:** *Histoplasma capsulatum*, Taiwan, galactomannan, endemic fungi, dimorphic fungi

## Abstract

Histoplasmosis is a global infection caused by the thermally dimorphic fungus, *Histoplasma capsulatum* complex. It is endemic in the United States, as well as in Central and South America. In Taiwan, histoplasmosis is rare, with the first reported case not occurring until 1977. We summarized a total of 17 cases reported in Taiwan over the past 40 years and provided detailed descriptions for four probable indigenous cases. Due to the lack of rapid diagnostic tools and clinical suspicion, histoplasmosis may be underdiagnosed in Taiwan. We recognize that a limitation of our review is the lack of data on the environmental surveillance for *H. capsulatum* complex in Taiwan. Conducting a further phylogenetic analysis on both environmental and clinical isolates would provide valuable evidence for the region.

## 1. Introduction

Histoplasmosis is among the most common endemic mycoses in the Americas; yet, it remains underdiagnosed and continues to pose clinical, diagnostic, and public health challenges [1]. The first human record of histoplasmosis dates back to 1905 during the construction of the Panama Canal by Samuel Darling. During the autopsy of the patient, an intracellular organism was found in the tissues. The organism was then named *Histoplasma capsulatum*. The infection of *H. capsulatum* can cause a wide range of presentations, including asymptomatic infections, acute pulmonary infection, chronic pulmonary infection, and disseminated disease [1,2]. There are three distinct varieties within the genus *Histoplasma*: the human pathogens, *Histoplasma capsulatum* var. *capsulatum* and *Histoplasma capsulatum* var. *duboisii,* and the horse pathogen *Histoplasma capsulatum* var. *farciminosum.* The *Histoplasm capsulatum* var. *capsulatum* is endemic to southern and northern Africa and *Histoplasma duboisii* var. *duboisii* is endemic to central and western Africa [3]. However, recent studies have revealed that the genus *Histoplasma* comprises a complex of cryptic species with several genetically distinct groups, including five genetic clusters: Nam 1 (North America 1), Nam 2 (North America 2), LAmA (Latin America), Panama, and Africa (formally known as *H. capsulatum* var. *duboisii*). The independently evolving lineages were named *H. capsulatum sensu stricto* when referring to the Panamanian lineage (H81 lineage), *H. mississippiense* (NAm 1), *H. ohiense* (NAm 2), and *H. suramericanum* (LAm A) [4]. Histoplasmosis in North America is caused by *H. mississippiense* and *H. ohiense*, while, in Latin America, the disease is caused by *H. suramericanum*, *H. capsulatum sensu stricto,* and other cryptic genotypes [5]. Since *H. capsulatum* is considered a complex of cryptic species, we used the term of *H. capsulatum* complex in the text below.

Over the last half-century, histoplasmosis has been increasingly diagnosed outside its historical geographic distribution. The variables affecting the epidemiologic and geographic changes are likely multifactorial and may include climate change and other anthropogenic activities. Current efforts are underway to identify the causes of these epidemiological changes [6]. Recognizing the expanded geographic distributions for histoplasmosis diagnoses is important in order to maintain a high clinical suspicion of these pathogens. *H. capsulatum* complex can be found in microfoci, particularly in soil containing bird excreta or bat guano [7]. Infection arises from occupational exposures such as cleaning chicken coops, building demolition and construction, farming, or outdoors activities like spelunking, all of which disrupt histoplasma habitats and result in the inhalation of microconidia [8,9]. An outbreak of severe histoplasmosis among tunnel workers has been reported in the Dominican Republic, leading to three deaths [10]. Implementing occupational health precautions during higher-risk activities can help reduce worker exposure.

*H. capsulatum* complex is a thermally dimorphic fungus, existing in a mycelial form in the environment and in a yeast form at 37 °C or higher. There are two types of conidia in the mycelia phase in the environment: macroconidia, measuring 8–14 μm, and microconidia, measuring 2–5 μm in diameter [2]. The micronidia, the infectious form, can be aerosolized to reach alveolar spaces, where they are engulfed by neutrophils and macrophages [11]. Then, the microconidia of the mycelia phase convert to the yeast form in alveolar macrophage [12]. Macrophages are able to migrate to other organs. Subsequently, the yeasts can cause the death of host cells, allowing them to migrate to another phagocytes [13]. The average incubation period is 1–3 weeks.

Despite its clinical and public health importance, the research on histoplasmosis is still limited. Taiwan is not regarded as an endemic region for histoplasmosis and the majority of recorded cases have been imported, corresponding to people who have been exposed to the fungus in endemic areas. In a study of histoplasmin skin tests conducted in the 1950s in Taiwan, only 7 (0.19%) of 3589 schoolchildren tested positive [14]. The prevalence rate of histoplasmosis was 0.24 per 100,000 (57 cases) according to national insurance data in Taiwan in 2013. However, due to limitations in the research data, the accuracy of diagnosis might be suboptimal, and the detailed information on the cases was unavailable [15]. To date, only case reports have been published. The results suggest that histoplasmosis could be scarce in Taiwan. Herein, we have summarized a total of 17 cases reported between 1977 and 2023 in Taiwan to raise awareness of histoplasmosis in a non-endemic area.

## 2. Materials and Methods

We carried out a literature search for relevant articles until 31 December 2023 in PubMed, Google Academic engines, and Embase with the following terms: “*Histoplasma capsulatum*”, “Histoplasmosis”, and “Taiwan”. Our review encompassed English literature and domestic publications from Taiwan. To investigate human infection caused by *H. cpasulatum* complex in Taiwan, all cases of human histoplasmosis were collected without any limitations related to age, sex, ethnicity, nationality, or type of study (Supplementary Figure S1).

### 2.1. Data Collection and Diagnosis of Disease

All literature and tables were reviewed for clinical details. A standarized form for data collection was designed. Data collected included age, sex, year of diagnosis or reporting, clinical manifestations, diagnostic methods, underlying diseases, treatment, and outcomes. In this article, we also review the literature regarding the diagnosis and treatment of histoplasmosis, focusing on *Histoplasma capsulatum* var. *capsulatum* (*H. capsulatum*).

Due to the lack of histoplasmosis serologic tests, antigen detection, and molecular methods in Taiwan, a confirmed diagnosis was typically achieved through cultures or histopathologic findings of specimens. In our review, pulmonary histoplasmosis was usually confirmed by cultures of sputum, lung tissue, or bronchial washing specimens, or by biopsy of lung tissue. Central nervous system histoplasmosis was diagnosed by cerebrospinal fluid culture. Disseminated histoplasmosis refers to the spread of *H. capsulatum* complex from the lungs through the blood to other organ systems, such as lymph nodes, gastrointestinal tract, or bone marrow. According to World Health Organization, disseminated histoplasmosis among people living with human immunodeficiency virus shoud be diagnosed by detecting circulating Histoplasma antigens [16], which is not available in Taiwan. The term disseminated histoplasmosis can also refer to the relentless growth of the organisms in multiple organ systems [17]. In our review, most cases of disseminated histoplasmosis were diagnosed by blood, bone marrow, or lymph nodes cultures, or positive result of cultures from more than one organ systems.

### 2.2. Risk Factors for Histoplasmosis

In our review, we particularly emphasized the risk factors for histoplasmosis infection as outlined in the literature. We documented patients’ immunocompromised status or their use of immunosuppressant medications. For those with human immunodeficiency virus infection, we recoreded the CD4 count whenever available. Additionally, we recorded patients’ exposure histories, encompassing their occupations and other activitis. Travel histories, especially to endemic regions abroad, were also recorded.

## 3. Results

The total 17 cases comprise 14 men and 3 women, with ages ranging from 23 to 86 years. Among the 17 cases, there were three Chinese participants and one Vietnamese participant. Nine cases had a travel history to endemic regions and the most common places were Southeast Asia and China (Table 1). Four cases were documented as having no overseas travel history, which suggests these patients may be indigenous cases in Taiwan.

Twelve patients had disseminated histoplasmosis, three had pulmonary histoplasmosis, one had central nervous system histoplasmosis, and one had bilateral adrenal histoplasmosis. The three most common underlying diseases of infected cases were human immunodeficiency virus (HIV) infection (41.1%), rheumatoid arthritis (11.8%), and diabetes mellitus (11.8%). Six cases of the HIV infection were diagnosed as acquired immunodeficiency syndrome (AIDS). Among the five cases of AIDS with available data, the CD4 counts were all ≦100 cells/μL. A comparison between the HIV and non-HIV groups showed no significant differences in genders, clinical manifestations, diagnostic methods, treatment, and outcomes. However, the patients with HIV infection were significantly younger than those without HIV (*p* = 0.006) (Supplementary Table S1).

Three cases (17.6%) were diagnosed by histopathology, four (23.5%) by culture, and ten (58.8%) by both histopathology and culture. Voriconazole was initially administered to two patients (11.8%), amphotericin B was administered to nine patients (52.9%), and liposomal amphotericin B was administered to one (5.9%) patient. A total of nine cases (52.9%) died of histoplasmosis.

### Probable Indigenous Cases

The first case (case 1 in Table 1) of histoplasmosis in Taiwan was not reported until 1977, a time when HIV examination was not available in Taiwan. A 32-year-old man, without any known underlying diseases, presented with symptoms including cough, backache, neck lymphadenopathy, anorexia, and weight loss. The laboratory results revealed leukocytosis, while chest radiography showed enlarged hilar lymph nodes. The patient was diagnosed with disseminated histoplasmosis based on histopathologic findings from a cervical lymph node biopsy specimen. The patient underwent anti-tuberculosis therapy and succumbed to histoplasmosis [18]. The second case (case 8 in Table 1) was a 78-year-old man with underlying rheumatoid arthritis. Before the onset of symptoms, he took oral hydroxychloroquine (400 mg/day), sulfasalazine (2000 mg/day), prednisolone (10 mg/day), and methotrexate (15 mg/week) for 4 months. He presented with generalized weakness and poor appetite for several weeks. Fever, icteric sclera, and multiple ecchymoses were also founded during presentation. The laboratory test revealed anemia, thrombocytopenia, and the elevation of total bilirubin and alkaline phosphatase. Refractory thrombocytopenia and the presence of young blood cells on the peripheral blood smears were founded. Chest radiography showed bilateral interstitial micronodules and a fibrocalcified pattern. The diagnosis of disseminated histoplasmosis was made on the basis of the histopathology and fungal culture of bone marrow. The micro-organism was confirmed by a polymerase chain reaction (PCR) assay. The patient’s condition improved after intravenous amphotericin B, and he was discharged with oral itraconazole. The itraconazole was discontinued after a 2-week therapy period due to impaired liver function. There was no relapse of histoplasmosis observed after stopping itraconazole for 3 months [25].

The third case (case 13 in Table 1) was a 23-year-old man who presented with a 2-week fever. Accompanying symptoms included cough, abdominal fullness, and weight loss. Laboratory tests showed thrombocytopenia and chest radiography revealed a cavity in the left upper lung zone. He denied traveling to foreign countries before admission. After admission, HIV infection was diagnosed. Antiretroviral therapy was immediately prescribed after diagnosis due to suspected virus-associated hemophagocytic syndrome. Bone marrow studies revealed yeast-like micro-organisms in histiocytes and evidence of hemophagocytosis. The serum galactomannan test result was also positive. Subsequently, both the blood culture and bone marrow culture yielded *Histoplasma* spp. The patient’s condition improved after treatment with liposomal amphotericin B, followed by a switch to oral voriconazole [30].

The fourth case (case 15 in Table 1) was a 46-year-old woman with systemic lupus erythematosus on hydroxychloroquine, azathioprine, prednisolone (5 mg/day), and methotrexate. She presented with a 20-day fever with lung infiltrates on the chest radiography. However, she did not have symptoms of the respiratory tract. The patient was treated as having community-acquired pneumonia at first but did not respond well. Laboratory tests revealed leukocytosis, bandemia, and thrombocytopenia. The chest computed tomography (CT) that followed indicated bilateral diffuse tiny lung nodules, interstitial thickening, and posterior lung consolidations. Hypoxemia developed after hospitalization. The peripheral blood smear showed neutrophils containing intracellular yeast-like micro-organisms. The bone marrow study indicated granulomas and hemophagocytosis with intracellular fungus. A lung tissue biopsy revealed ovoid-shaped micro-organisms on the Gomori methenamine silver stain. Cultures of lung and skin tissue yielded *H. capsulatum* which was confirmed by PCR. The patient received intravenous amphotericin B for two weeks, followed by itraconazole for one year. However, she experienced a recurrence of histoplasmosis after discontinuing itraconazole for three months. Dyspnea and fever developed and she was admitted to the intensive care unit due to respiratory distress. Empiric amphotericin B was given. The bone marrow studies revealed a fungal infection and the bronchoalveolar lavage culture yielded *H. capsulatum*. She was discharged with itraconazole. This woman was a housewife with no experience of traveling abroad. However, she was involved in the renovation of a chicken farm before the onset of symptoms, which may be relevant since *H. capsulatum* complex is primarily found in soil containing a high concentration of bird excrement [32].

## 4. Discussion

Histoplasmosis is endemic throughout Central and South America, as well as in the United States. Within the United States, it is primarily centered in the midwestern and central regions, along with the Ohio and Mississippi River Valleys. A recent study indicated that over a third of individuals in Central and South America have been exposed to Histoplasma, particularly those with AIDS, solid organ transplantation, or other diseases associated with impaired T cells [7]. Besides America, histoplasmosis has been reported in China, central Myanmar, southern Thailand, northern Philippines and parts of Indonesia, and India [1]. In China, 75% of the reported cases occurred within the Yangtze River basin. The prevalence of Histoplasmin skin test reactivity ranges from 6% to 50% [35]. In Europe, the majority of autochthonous cases of histoplasmosis have been identified in Italy [36]. In non-endemic areas, imported histoplasmosis due to migration is increasing and should not be neglected.

Histoplasmosis is not commonly found in Taiwan, and some cases develop histoplasmosis with a remote travel history to endemic areas. Since reactivation does not appear to be the predominant pathogenesis of histoplasmosis [37], it is not certain whether these patients had imported histoplasmosis or were indigenous cases. The four probable indigenous cases were identified based on their self-reported absence of travel history. To date, there has been no environmental surveillance for H. capsulatum complex in Taiwan. Notably, two patients (case 15 and 16) among the 17 cases reported a history of exposure to a chicken farm. Performing a phylogenetic analysis on both environmental and clinical isolates could potentially offer valuable evidence regarding the endemicity of H. capsulatum complex in the region.

The manifestations of histoplasmosis can vary widely, ranging from asymptomatic to life-threatening, depending on the individual’s immune status and the extent of the infection. Interferon-γ released by activated macrophages and CD4-T lymphocytes mediated the cell-medicated immune response to H. capsulatum complex [38]. Nevertheless, in individuals with HIV infection, macrophages fail to mount an effective immune response [11]. The progressive impairment of the cellular immunity of advanced HIV causes disseminated infections with H. capsulatum complex. Tumor necrosis factor antagonists [39], calcineurin inhibitors [40], and monoclonal antibodies [41] that reduce the number or function of T-cells also raise the risk of disseminated histoplasmosis. The majority of primary infections are asymptomatic or mild, and a small proportion of individuals develop acute pulmonary histoplasmosis, which may be accompanied by rheumatologic symptoms. Rare cases of acute histoplasmosis may lead to pericarditis. Chronic cavitary pulmonary histoplasmosis is often associated with risk factors such as smoking or chronic lung disease. In older and immunosuppressed patients, primary histoplasmosis may advance to progressive disseminated histoplasmosis (PDH), which has a high mortality rate. Central venous system involvement may occur in some cases of PDH [1,2].

In our review, six patients with histoplasmosis had AIDS and one had HIV infection. The patients’ age ranged from 23 to 55 years old and all were male. Five cases with available data had CD4 counts ≦100 cells/μL. Among the seven cases, six (86.7%) presented with disseminated histoplasmosis, while one (14.3%) had central nervous system involvement. Both of these manifestations are commonly associated with advanced HIV infection. At least four cases resulted in death due to histoplasmosis. Histoplasmosis is a worldwide opportunistic infection in patients with HIV infection and may be considered an AIDS-defining illness. Patients with CD4 counts of <150 cells/μL are at the highest risk for histoplasmosis [42]. In countries with access to antiretroviral therapy, the incidence of histoplasmosis has declined dramatically. Nevertheless, histoplasmosis remains life-threatening in many resource-limited regions.

Patients without recognized immunocompromising conditions or not taking immunosuppressant medication should consider assessing their anti-interferon-γ autoantibodies. Interferon-γ is vital for macrophage-mediated killing and granuloma formation against intracellular pathogens like Mycobacterium, and Histoplasma. In a previous investigation conducted in Thailand, 27 out of 74 patients (36.5%) with an anti-interferon-γ presentation had other opportunistic infections besides nontuberculous mycobacteria, including disseminated Salmonella, Histoplasma, and Cryptococcus [43]. Case 5, as depicted in Table 1, had both invasive salmonellosis and histoplasmosis. Anti-interferon-γ autoantibodies should be considered due to the potential for impaired cellular-mediated immunity [22].

A variety of laboratory tests, including fungal culture, histopathology, cytology, serology, antigen detection, and molecular testing, can be used to diagnose histoplasmosis [1]. The gold standard for diagnosing histoplasmosis is still isolating the mold from specimens, such as blood, bone marrow, sputum, or bronchoscopy specimens. However, this process requires prolonged incubation, typically within 3 weeks, but it may take up to 8 weeks. At 25–30 °C on Sabouraud’s dextrose agar, the colonies of the thermally dimorphic fungus exhibited a white, suede-like cottony texture with a pale yellow-brown reverse. With lactophenol cotton blue staining, the microscopic morphology reveals the presence of rounded, single-celled, 8–14 μm-diameter, tuberculated macroconidia formed on conidiophores. If incubated at 37 °C, the mold would undergo a transformation into the yeast phase [1,44].

The pathologic feature on histologic examination is the presence of granulomas, which may be caseating or noncaseating. Stains for the presence of H. capsulatum complex may rapidly identify the fungus in various tissues. Specimens stained with Gomori methenamine silver nitrate may show the characteristic oval yeasts, measuring 2–4 μm, with narrow-based budding [45]. According to international guidelines, the histopathologic examination of a specimen revealing the presence of yeast cells, further confirmed with the amplification of fungal DNA by PCR combined with DNA sequencing, consistent with H. capsulatum complex, is also the diagnostic standard for proven histoplasmosis [46].

The main serologic tests for the detection of H. capsulatum complex antibodies are immunodiffusion, complement fixation, and enzyme immunoassay. Antibodies can be detected 4–6 weeks after exposure and persist for years. Serologic tests may have limited usefulness in the diagnosis of acute histoplasmosis and a poorer sensitivity in immunocompromised patients due to impaired antibody responses [45,47]. The detection of antigenemia and antigenuria with a quantitative assay enzyme-linked immunosorbent assay provide a rapid method for diagnosing histoplasmosis [48]. It can also be performed on bronchoalveolar lavage and cerebrospinal fluid specimens. The sensitivity is higher in severe disease or immunocompromised individuals due to the higher burden of infection. Monitoring the decline in antigen level can also serve as an indicator of effective treatment [49].

In Taiwan, most histoplasmosis are diagnosed or suspected by culture and histopathology, and then further confirmed by PCR. The absence of rapid diagnostic tools could lead to the high percentage of disseminated histoplasmosis in Taiwan. The higher rate of disseminated histoplasmosis may also explain the high positive rate of culture in these patients.

Galactomannan (GM) is a polysaccharide cell component of Aspergillus species, as well as a component of the cell walls of other fungi, including Histoplasma, Penicillium, Blastomyces, etc. Patients with positive histoplasmosis antigen tests can have false-positive Aspergillus GM tests in the serum, urine, or bronchoalveolar lavage (BAL) due to cross-reactivity [50]. One of the reviewed histoplasmosis cases was diagnosed in our hospital (case 16 in Table 1), with an optical density index of 0.67 for BAL fluid and 6.7 for serum GM [33]. The GM test has also been employed as a surrogate biomarker for the evaluation of antifungal response [34]. However, high concentrations of Histoplasma antigen may be required for cross-reactivity [51]. The range (1.2–8.61, OD Index) of serum GM test levels for culture-proven Histoplasma infections varied according to the previous literature [52]. In Taiwan, diagnostic tools such as the serologic test and antigen test for histoplasmosis are unavailable. The GM assay might serve as an adjuvant for the consideration of histoplasmosis.

Itraconazole and lipid formulation amphotericin B are both recommended for the treatment of histoplasmosis, depending on the site of infection and the severity of the disease. Newer triazole antifungals, including voriconazole and posaconazole, are considered to have fungicidal activity against Histoplasma. Among the 17 cases, voriconazole was initially administered to two patients; however, poor clinical responses were observed. One patient succumbed, and the other had to be switched to liposomal amphotericin B due to a suboptimal response. In a retrospective cohort study of adult patients diagnosed with histoplasmosis, patients receiving voriconazole (*n* = 19) were contrasted with those receiving itraconazole (*n* = 175). The study, with balanced baseline characteristics between the two groups, revealed similar 180-day mortality rates in both the voriconazole group (31.6%) and the itraconazole group (23.4%). However, a statistically significant association of voriconazole with increased early mortality (0 to 42 days) was observed (adjusted hazards ratio, 4.3; 95% confidence interval, 1.3–13.9) [53]. Due to the limited evidence on their effectiveness, newer azoles are not recommended as first-line agents for patients with moderate or severe histoplasmosis. Further investigations are warranted to elucidate the role of newer azoles in the treatment of histoplasmosis.

## 5. Conclusions

In Taiwan, the absence of quick diagnostic tools and physicians’ familiarity with histoplasmosis may result in underdiagnosis and high mortality. For immunocompromised patients at risk of environmental exposure, or an elevation in the GM test without evidence of Aspergillus infection, histoplasmosis should be suspected even in non-endemic areas. A further environmental investigation for H. capsulatum complex would help us to better understand the epidemiology in Taiwan.

## Figures and Tables

**Table 1:** Characteristics of cases of histoplasmosis reported in Taiwan.

No./ Ref	Age/ Sex		Diagnosed or Reported Year	Manifestation	Underlying Disease	Travel History	Diagnostic Methods	Initial Antimicrobial Treatment	Outcome
1/[18]	32/M		1977	Disseminated histoplasmosis	None ^a^	None	Lymph node biopsy	Anti-TB drugs	Died
2/[19]	77	M	1994	Laryngeal histoplasmosis	Old pulmonary TB, Addison’s disease under lifelong corticosteroid treatment	Europe, Indonesia, China, Saudi Arabia	Laryngeal biopsy and fungal culture	Ketoconazole	Died
	86		2003	Pulmonary histoplasmosis			Sputum culture and lung biopsy	AmB	
3/[20]	37/M		1996	Disseminated histoplasmosis	AIDS (CD4 count: NA), old pulmonary TB, cerebral toxoplasmosis	Myanmar	Skin, bone marrow, liver biopsy, and fungal culture	Fluconazole	Died
4/[21]	27/M		1997	Disseminated histoplasmosis	AIDS (CD4 count: 2/μL)	Chinese, Thailand, Malaysia, Singapore	Colon tumor biopsy and fungal culture	AmB	Survived
5/[22]	46/M		1999	Disseminated histoplasmosis	Concomitant nontyphoid salmonellosis	Indonesia	Skin biopsy and skin, blood, and pleural fungal culture	AmB	Died
6/[23]	30/M		2000	Disseminated histoplasmosis	AIDS (CD4 count: 76/μL)	Unknown, but he was a sailor	Stomach and lymph node biopsy	AmB	Died
7/[24]	55/M		2004	CNS histoplasmosis and disseminated TB	Chronic renal insufficiency, AIDS (CD4 count: 2/μL)	Chinese Myanmar, China	CSF fungal culture	AmB	Died
8/[25]	78/M		2006	Disseminated histoplasmosis	Rheumatoid arthritis	None	Bone marrow aspiration biopsy and culture; confirmed by PCR	AmB	Survived
9/[26]	37/M		2008	Pulmonary histoplasmosis	None	Chinese Myanmar	Lung biopsy	Levofloxacin	Survived
10/[27]	65/F		2008	Disseminated histoplasmosis	Hemolytic anemia	Not mentioned	Bone marrow biopsy and culture	AmB	Died
11/[28]	31/M		2011	Disseminated histoplasmosis	AIDS (CD4 count: 15/μL)	Vietnamese Vietnam	Bone marrow, gingiva, lymph node, nasopharynx biopsy; fungal culture of gingival biopsy	Not mentioned	Not mentioned
12/[29]	74/M		2012	Bilateral adrenal histoplasmosis	Diabetes mellitus	Not mentioned	Adrenal gland biopsy and culture	AmB	Survived
13/[30]	23/M		2017	Hemophagocytic syndrome with disseminated histoplasmosis	Acute human immunodeficiency viral infection (CD4 count: NA)	None	Blood and bone marrow cultures	Liposomal AmB	Survived
14/[31]	37/M		2017	Disseminated histoplasmosis	AIDS (CD4 count: 0/μL)	Not mentioned	Blood culture and bone marrow biopsy; confirmed by PCR assay	Not mentioned	Died
15/[32]	46/F		2018 and recurrence in 2019	Recurrent disseminated histoplasmosis	Systemic lupus erythematosus	None (Occupation: chicken farmer)	Lung biopsy; lung tissue, skin biopsy culture; confirmed by PCR	AmB	Survived
16/[33]	74/M		2022	Disseminated histoplasmosis	Diabetes mellitus	Southeast Asia and China decades ago for a few days	Blood, ascites, bone marrow culture; confirmed by the sequence of internal transcribed spacers of ribosomal DNA	Intravenous voriconazole	Died
17/[34]	83/F		2022	Pleuropulmonary histoplasmosis	Rheumatoid arthritis	A remote travel history to Thailand, the US, and European countries	Pleural effusion, lung tissue, mediastinal lymph node, and bronchial washings culture	Voriconazole, and then change to liposomal AmB	Died of natural causes 3 months later

Abbreviation: Ref, reference; M, male; F, female; TB, tuberculosis; AmB, amphotericin B; AIDS, acquired immunodeficiency syndrome; NA, data not available; CNS, central nervous system; CSF, cerebrospinal fluid; PCR, polymerase chain reaction. DNA, desoxyribonucleic acid. ^a^ HIV examination was not available in Taiwan until 1980s.

## Data Availability

The data are contained within the article.

## Supplementary Materials

The following supporting information can be downloaded at: https://www.mdpi.com/article/10.3390/life14060738/s1, Table S1. Characteristics of patients with histoplasmosis reported in Taiwan. Figure S1. Flowchart for the identification of eligible studies.
