# Supplementary material for: Histoplasmosis in Taiwan: Case Summary and Literature Review
Source: Life (Basel). 2024 Jun 7;14(6):738. doi: 10.3390/life14060738 (PMC11204960; doi:10.3390/life14060738)
Supplement: Supplementary file 1 [file life-14-00738-s001.zip › life-2989496-supplementary.pdf]

Supplementary material

Table S1. Characteristics of patients with histoplasmosis reported in Taiwan

Variable	Overall (n =17)	HIV (n=7)	Non-HIV (n =10)	p Value
Demographic features				
Median age, year (range)	46 (23-86)	31 (23-55)	69.5 (32-86)	0.006
Female, no. (%)	3 (17.6)	0 (0)	3 (30)	0.228
Indigenous case, no. (%)	4 (23.5)	1 (14.3)	3 (30)	0.603
Treatment, no. (%)				
Amphotericin B	9 (52.9)	3 (42.9)	6 (60)	0.637
Liposomal amphotericin B	1 (5.9)	1 (14.3)	0	0.412
Voriconazole	2 (11.8)	0	2 (20)	0.485
Fluconazole	1 (5.9)	1 (14.3)	0	0.412
No antifungal treatment	2 (11.8)	0	2 (20)	0.485
Not mentioned	2 (11.8)	2 (28.6)	0	0.154
Death due to histoplasmosis, no. (%)	9 (52.9) (n=16)	4 (57.1) (n=6)	5 (50)	0.633
Manifestations, no. (%)				
Pulmonary histoplasmosis	3 (17.6)	0	3 (30)	0.228
CNS histoplasmosis	1 (5.9)	1 (14.3)	0	0.412

Adrenal histoplasmosis	1 (5.9)	0	1 (10)	>0.99
Disseminated histoplasmosis	12 (70.6)	6 (86.7)	6 (60)	0.338
Median CD4 counts, range (cells/μL)		2 (0-76) (n=5)		
Diagnostic methods, no. (%)				
Culture	4 (23.5)	2 (28.6)	2 (20)	>0.99
Histopathology	3 (17.6)	1 (14.3)	2 (20)	>0.99
Both culture and histopathology	10 (58.8)	4 (57.1)	6 (60)	>0.99

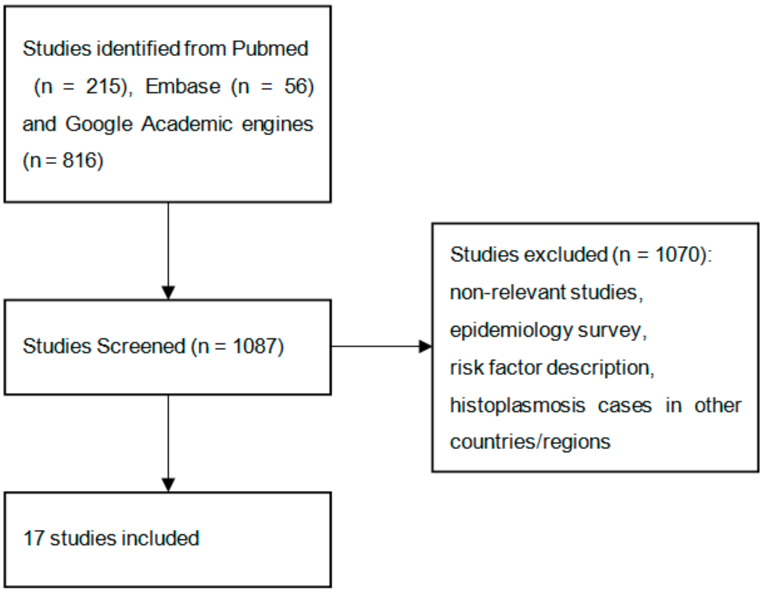

Figure S1. Flowchart for the identification of eligible studies
